# Parry Romberg Syndrome in a Young Ghanaian: A Case Report and a Literature Review

**DOI:** 10.7759/cureus.32287

**Published:** 2022-12-07

**Authors:** Klenam Dzefi-Tettey, Emmanuel K Edzie, Mark-Young Seadey, Edmund K Brakohiapa, Samuel Asiamah, Simpson K Mensah, Kafui K Kekeshie, Caroline E Ntiamoah-Koufie, Denisson K Agala, Franklin Acheampong

**Affiliations:** 1 Department of Radiology, Korle-Bu Teaching Hospital, Accra, GHA; 2 Department of Medical Imaging, University of Cape Coast, Cape Coast, GHA; 3 Departments of Dermatology, Internal Medicine and Therapeutics, University of Ghana Medical School, Accra, GHA; 4 Department of Radiology, University of Ghana Medical School, Accra, GHA; 5 Department of Radiology, Korle Bu Teaching Hospital, Accra, GHA; 6 Department of Medical Imaging, University of Health and Allied Sciences, Ho, GHA; 7 Department of Radiology, Tema General Hospital, Tema, GHA; 8 Research Unit, Korle Bu Teaching Hospital, Accra, GHA

**Keywords:** computed tomography scan, ghana, magnetic resonance imaging, progressive hemifacial atrophy, parry romberg syndrome

## Abstract

Parry Romberg syndrome (PRS), also known as progressive hemifacial atrophy, is a very rare self-limiting disease, which affects the skin and subcutaneous tissues, underlying musculature, cartilage, and bony structures of one half of the face with a resultant hemiatrophy and alopecia areata. It presents in children and young adults, with a slow progression of the atrophy for several years, and then becomes stable. Magnetic resonance imaging (MRI) or computed tomography (CT) scan of the cranium demonstrates the radiological feature of hemiatrophy very clearly. We report a case of PRS in a nine-year-old girl with characteristic features which was diagnosed based on medical history, clinical signs, and radiological findings on cranial CT scan and MRI.

## Introduction

Parry Romberg syndrome (PRS) is a rare disease, with a prevalence of at least one in 700,000 births. The initial sign is thinning of the cutaneous and subcutaneous tissues, which is often preceded by alopecia areata. It involves the nose, mouth, or ear, and damage is usually limited to the distribution of the trigeminal nerve. These lesions do not cross the midline and craniofacial asymmetry results, which causes esthetic discomfort, thus reducing self-esteem and affecting the growth and intellectual development of the individual [1-3].

Parry was the first to describe this condition in 1825 followed by Romberg in 1846. In 1871, Eulenberg named it progressive hemifacial atrophy [2]. It presents in young adults and children and has a male-female ratio of 1:2.23. It typically manifests between five and 15 years and progresses slowly over a variable time course. It is self-limiting, and not usually fatal but neurological symptoms may develop [1,4,5]. The pathophysiology is poorly understood [5-7].

Magnetic resonance imaging (MRI) or computed tomography (CT) scan of the head reveals varying degrees of bone and soft tissue atrophy on the affected side. Another common neuroimaging finding is white matter hyperintensities on T2 and fluid-attenuated inversion recovery (FLAIR) sequences on brain MRI and intracranial calcifications on CT. Advanced neuroimaging and metabolic/functional techniques like diffusion tensor imaging (DTI) and magnetic resonance spectroscopy (MRS) can also be utilized. MRS can also show abnormal spectral patterns and decreased N-acetyl aspartate level in the affected region of the brain and DTI, and a fractional anisotropy mapping reveals a decrease in anisotropy of the water molecules in the white matter of the affected area [6,8-11].

## Case presentation

A nine-year-old girl with a provisional diagnosis of PRS was referred to the radiology department for a CT scan of the head. She is the first-born of three siblings who are all healthy. No known family member has abnormal features. Her antenatal, peri-natal, and post-natal periods were uneventful. She was born by spontaneous vaginal delivery, cried at birth, and her birth weight was 3.0 kg. She had no relevant previous medical history. She had normal developmental milestones and is currently in school and doing well academically. She weighed 37 kg with a height of 1.39 m. Routine blood investigations were all within normal limits.

**Figure 1:**
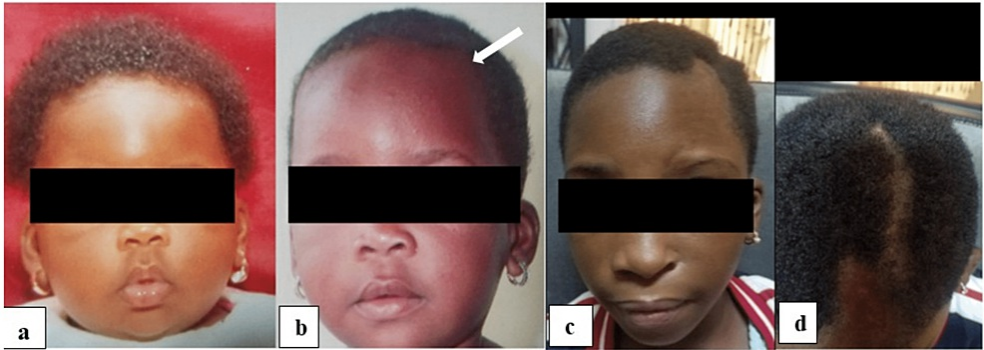
A child with Parry Romberg syndrome with (a) and (b) showing normal facial appearance (no facial asymmetry); however, (b) shows the beginning of hair loss and subtle hypopigmentation of the left frontal scalp (white arrow); (c) and (d) show hypopigmentation, progressive left facial hemiatrophy, small left nostril, loss of hair of the left eyebrow, and alopecia areata

Her mother complained that from age four, there was a gradual progressive reduction in the size of her face on the left side, hypopigmentation of the skin of the frontal scalp, and ipsilateral loss of hair which preceded the hemifacial atrophy. On physical examination, there was left facial hemiatrophy resulting in a small nasal cavity, a depression of the left frontal skull, alopecia areata, hypopigmentation of the ipsilateral skin of the frontal scalp, and eyebrow alopecia. There was no tongue atrophy, and she was a very active girl with no neurological deficits. Her complaints were nasal congestion, occasional headaches, anxiety about her facial asymmetry, and constant questioning from her classmates. Figure 1 shows the mild progressive course of the disease.

**Figure 2:**
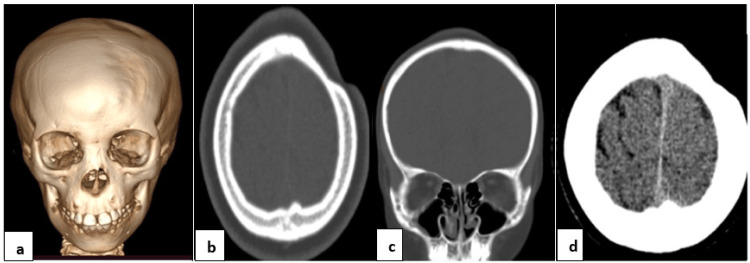
Head CT scan 3D volume rendering (a), axial bone window (b), coronal bone window (c), and axial brain window (d) showing thinning of the left frontal and parietal bones, subcutaneous tissues/fat, and reduction in the left nasal cavity. Subtle effacement of the sulci of the left frontal lobe is also seen. CT, computed tomography; 3D, three-dimensional.

Ultrasound scans of the parotid and submandibular glands were unremarkable. A head CT scan was done without a contrast medium (Figure 2).

**Figure 3:**
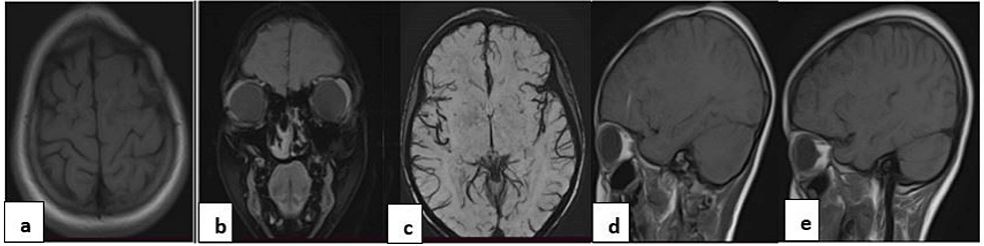
MRI of the head, axial unenhanced T1W image, showing subtle effacement of the sulci of the left frontal lobe and thinning of the ipsilateral subcutaneous scalp tissues (a), coronal T1W image post gadolinium enhancement showing a marked reduction in the left nasal cavity and subtle effacement of the sulci (b), axial SWI revealed no micro hemorrhages or aneurysms (c), the left para sagittal MRI (d) also showed thinning of the subcutaneous frontal scalp tissues and subtle frontal lobe atrophy compared with the contralateral right parasagittal image (e). MRI, magnetic resonance imaging; T1W, T1-weighted; SWI, susceptibility-weighted imaging.

 MRI with gadolinium was also done using a 1.5 Tesla MRI machine (Figure 3).

The patient and her family were counseled and symptomatic treatment was given. She is being followed up.

## Discussion

PRS is a rare neurocutaneous disease of sporadic occurrence, which is characterized by a one-sided self-limiting facial atrophy [1]. The distribution of the atrophy is often unilateral (limited to one side of the face) and usually on the left side as seen in our patient. Ipsilateral involvement of the trunk and limb is rare; however, 20% of cases were described as being bilateral [6,7,11,12]. Guerrerosantos et al. classified the syndrome into four types, namely types 1, 2, 3, and 4 tissue depressions. Types 1 and 2 show mild involvement of the soft tissues of the face and the skin, while types 3 and 4 are severe diseases with involvement of bone and cartilage. Patients also have severe functional problems, especially with the nose and lips [13,14]. This confirms why our patient experienced nasal congestion. This syndrome usually starts in the first two decades of life and this is corroborated by Jones et al., who reported three patients as young as an infant, a two-year-old, and a four-year-old. This was exhibited by our patient whose symptoms started at age 4 and she is now nine years old. PRS is also known to occur in the fifth or sixth decade of life and has a male-female ratio of 1:2.23 [1,4,5,11,12,14]. Our patient has female gender.

Clinically, the initial signs are thinning of the skin and subcutaneous tissues, usually preceded by alopecia areata, or vitiligo as seen in our patient [1,6]. The syndrome usually progresses slowly over a variable course from two to 20 years, reaching a “burned-out phase” and stabilizes [1,3,9,11,15]. Our patient is being followed up. The etiology of PRS is unclear and a theory suggests that it is an autoimmune disorder since patients have other autoimmune diseases like systemic lupus erythematosus, rheumatoid arthritis, and generalized myopathy. Others are vascular dysfunction, neuro-vasculitis, infectious etiology, and trauma. None of these conditions were seen in our patient.

The diagnosis of PRS is mostly a clinical one, aided by findings on histopathology and imaging. There are no accepted standardized diagnostic criteria; however, reported photographs of the individual should be kept and followed throughout the course of the disease and follow-up [9,11]. This is what we are doing for our patient, and comparing her photographs taken during infancy with the current state helped us in making the initial diagnosis. If overall clinical presentation progresses very fast, a biopsy of the skin may be considered and electroencephalogram (EEG) changes may also be seen in patients with seizures [9]. Our patient did not have any seizures. A high titer of a positive anti-nuclear antibody may be seen in 25-52% of patients. Some neuroimaging findings in PRS are cutaneous and subcutaneous tissues atrophy of the hemiface or body, cerebral atrophy, corpus callosum infarction, subcortical and deep white matter lesions, aneurysms, leptomeningeal and meningeal enhancement, and intracranial calcifications. Others are cortical thickening and dysgenesis, cystic degeneration, and hydrocephalus [9].

CT scan best demonstrates calcifications and bony changes of the hemicranium and facial skull while MRI is sensitive in identifying gliosis and atrophy. These abnormalities are usually non-enhancing, but iodinated contrast medium and gadolinium were useful in a few cases in showing areas of active inflammation on head CT and MRI, respectively [16]. Typical findings on MRI are hyperintense white matter lesions on T2-weighted and FLAIR sequences, which are distributed predominantly on the ipsilateral hemisphere but are sometimes bilateral. These hyperintense lesions usually do not progress despite progressive skin and skeletal changes [9-11]. Head CT scan and MRI in our patient, however, revealed thinning of the subcutaneous fatty tissues of the left hemiface, small nasal cavity, depression and thinning of the left frontal lobe, and subtle effacement of the sulci of the ipsilateral frontal lobe. Ultrasonography can identify sclerosis, and monitor the disease activity and progress of treatment by taking measurements of dermal thickness and noting the echo pattern of the affected sites. Color Doppler is also useful in detecting dermal blood flow, and increased flow suggests active disease. Increased blood flow and hypoechogenicity of the ipsilateral parotid and salivary glands also suggest inflammation and an active disease process [9]. Ultrasound of the parotid and salivary glands was unremarkable in our patient.

The differential diagnosis of PRS includes other forms of juvenile localized scleroderma, Rasmussen encephalitis, Barraquer-Simons syndrome, primary hemifacial hypertrophy, and congenital hemiatrophy. Others are congenital deformities such as “wry neck” fat necrosis, either from infection like bulbar poliomyelitis, connective tissue disease, or trauma [9,16-19]. There is no cure and surgical treatment requires a multidisciplinary team consisting of physicians, dermatologists, plastic surgeons, radiologists, dentists, and psychologists [9,11].

## Conclusions

This rare condition can easily be missed or misdiagnosed during a routine doctor-patient consultation, as it is often very subtle initially. The accurate and early diagnosis of PRS demands an in-depth knowledge of this condition among the practitioners who may be involved in the initial encounter with the patient and management. A detailed review of the patient’s medical history, previous photographs, and an extensive physical examination are required to look for signs of progressive loss of skin, fat, muscle, and bone. Patients with facial asymmetry may develop psychological problems; hence, clinical psychologists must be involved very early in the management. This case report documents the classic features and hence contributes to the understanding of the disease.
